# Effect of LPS on Cytokine Secretion from Peripheral Blood Monocytes in Juvenile Idiopathic Arthritis-Associated Uveitis Patients with Positive Antinuclear Antibody

**DOI:** 10.1155/2021/6691681

**Published:** 2021-05-03

**Authors:** Jing Wang, Huiru Wu, Xinli Liu, Huiyu Jia, Hong Lu

**Affiliations:** ^1^Department of Ophthalmology, Beijing Chaoyang Hospital, Capital Medical University, Beijing 100020, China; ^2^Department of Ophthalmology, The First Clinical Medicine College of Lanzhou University, Lanzhou 730000, China; ^3^Department of Ophthalmology, Haidian Maternal and Child Health Hospital, Beijing 100080, China

## Abstract

**Objectives:**

Antinuclear antibody (ANA) positivity is a key finding in JIA-associated uveitis (JIAU), but there are quite a few patients with negative ANA. There is no relevant report on the difference of their clinical manifestations. Previous animal model studies have found that the occurrence of uveitis is related to macrophage activation. In this article, our goal is to investigate changes in the morphology and cytokines of peripheral blood mononuclear cells (PBMCs) in uveitis patients testing positive or negative for ANAs after lipopolysaccharide (LPS) stimulation.

**Methods:**

A total of 30 patients were included in this study (10 in each group). They were divided into three groups (the ANA-positive [ANA+] group, ANA-negative [ANA-] group, and control group). There were ten patients (6 females and 4 males) in each group. Peripheral venous blood was collected into a heparinized tube, and PBMCs were isolated as soon as possible by the Ficoll-Hypaque density gradient separation method. Isolated cells were mixed with RPMI-1640 medium, and the cell concentration was adjusted to ensure that each patient had the same number of cells entering the study. After putting the extracted PBMC into the culture plate, LPS was added carefully to the plate. The cell culture supernatants were collected at 0 h, 3 h, 6 h, 12 h, and 24 h after LPS stimulation to detect the concentrations of IL-6, IL-1, TNF-*α*, and IL-10. Immunofluorescence was used to discover the deformation of macrophages after LPS stimulation.

**Results:**

The newly isolated cells were approximately round. 6 h after LPS stimulation, the ratio of noncircular cells/circular cells was the highest in the ANA+ group. Unlike IL-10 that has been rising during the observation period, IL-6, IL-1, and TNF-*α* peaked at 6 h after LPS stimulation.

**Conclusion:**

With LPS motivation, cytokines in the ANA+ group increased the most violently.

## 1. Introduction

Juvenile idiopathic arthritis (JIA) comprises a group of idiopathic arthritides that occur before the age of 16 years and persist for at least 6 weeks [1]. Clinical observation found that most uveitis occurred with oligoarthritis. Most individuals with JIA-associated uveitis (JIAU) exhibit asymptomatic anterior uveitis. With the significant improvement of diagnosis and treatment capabilities, the prognosis of JIAU has been greatly improved; however, the disease is still the main cause of visual impairment in children with uveitis [2]. In clinical practice, we found that most patients with an early onset age were antinuclear antibody (ANA) positive (ANA+). This was consistent with Kotaniemi et al.'s finding that ANA positivity was a risk factor for early onset of JIAU [3]. Since the ocular symptoms were not obvious at the beginning of the onset, most of the discomfort cannot be accurately expressed by the children; therefore, some patients with initial uveitis were easily overlooked. Repeated ocular inflammation can produce ocular complications, and patients with severe complications often have poor prognosis. Regular eye examinations on children are an effective way to detect diseases early. Although ophthalmologists and rheumatologists have a deeper understanding of the treatment of the disease, its pathogenesis is still unclear. Therefore, it is imminent to explore the pathogenesis of this disease in order to obtain better treatment.

The incidence of JIAU in North America and Europe appeared to be higher than that in Asia. Even in the same region, the incidence and disease subtypes vary from race to race. It was easy to infer that diversity in access to health care and knowledge of JIAU may affect reports of incidence in certain areas. However, the authors were more inclined to believe that the prevalence of this disease in regions and races was different [4].

JIAU is a type of chronic inflammation that mainly manifests as anterior uveitis, but frequent acute attacks have a great impact on the prognosis of the eye. Since children's health care is carried out by a special institution, we selected children who came to the hospital for ocular complications as our research objects. We found that most patients with an earlier age of onset had ANA+ and that had poorer vision. We inferred that the children could not express discomfort accurately and that parents' neglect of the disease might be the cause of this consequence. However, we believe that frequent acute attacks of inflammation which had not been properly controlled were the main reasons for ocular complications. In our previous studies on animal models of acute uveitis, we found that after LPS stimulation, a large number of inflammatory cells were produced in the anterior chamber. The inflammation signal pathway of the iris ciliary body tissue produces inflammatory factors [5–7]. Therefore, is there a difference in the response of ANA+ and ANA-negative (ANA-) patients to LPS stimulation? In this study, we isolated and cultured monocytes from ANA+ and ANA- patients to observe the changes in cytokines and cell morphology after LPS stimulation.

## 2. Materials and Methods

### 2.1. Study Population

JIAU were diagnosed jointly by an ophthalmologist and a pediatric rheumatologist. The subjects enrolled were patients who came to the Eye Clinic of Beijing Chaoyang Hospital from January to September of 2018. All participants, including the healthy control group, had no fever or other diseases recently. All patients in the group were in remission without systemic or ocular inflammatory activity. Rheumatologists recommended oral methotrexate to control arthritis. There was no eye medication, and no patients used biological agents such as adalimumab. Before starting the research, we asked parents to sign a written informed consent. The clinical research protocol was approved by the Ethics Committee of Beijing Chaoyang Hospital, Capital Medical University. In order to reduce the difference of gender, the proportion of girls and boys in this study was the same. The basic information was as follows: 4 males and 6 females in the ANA+ group, aged 2-16 years (average 8.6 years), comprising 3 cases of banded keratopathy, 4 cases of posterior synechiae, and 10 cases of cataract. (Table 1) The ANA- group included 4 boys and 6 girls, aged 7-16 years (average age: 11.2 years; 6 cases of posterior synechiae; 2 cases of glaucoma; 10 cases of cataract). The volunteers included 4 boys and 6 girls, aged 9-16 years (average age: 12.6 years) (Table 2). Due to insufficient health care of children in some areas, the onset age of systemic arthritis and ocular inflammation may have been earlier than that reported in the statistics. Thus, the onset age of some patients in this table was accurately described as the age at diagnosis.

### 2.2. Isolation of Monocytes

According to the method of Antonakos et al., peripheral blood was collected into the heparinized tube, and peripheral blood mononuclear cells (PBMCs) were separated using the Ficoll-Hypaque method as soon as possible [8]. 6 ml of peripheral blood venous was collected from each patient. The cell suspension was washed three times with PBS (wash off platelets, etc.), and PBMC was counted with trypan blue-eliminated dead cells. Then, the cells were suspended in RPMI-1640 evenly. Before the experiment, we adjusted the cell concentration to ensure the number of cells entering the study was the same for each subject. The extracted cell suspension was injected into a 24-well cell culture plate with an average concentration of 1 × 10^5^/ml. It was difficult for children to collect blood, especially the younger ones. Parents were not willing to let us collect more than 6 ml blood for experimental research. If the number of cells was insufficient, the immunofluorescence staining could not be performed well. So for each subject, we used one-third of the cell suspension for immunofluorescence staining. Limited by the amount of blood collected and in order to achieve a better presentation, we did not conduct fluorescence staining for all observed time points. Instead, a cytokine peak (6 h after LPS stimulation) was selected to observe the change in cell morphology with immunofluorescence. The remaining suspension was injected into the corresponding cell culture wells, and the cell culture supernatants were taken for cytokine detection. Each observation index was repeated 3 times.

### 2.3. Enzyme-Linked Immunosorbent Assays

The cell supernatants were harvested at different times (0, 3, 6, 12, and 24 h) after LPS stimulation (final concentration of 1 *μ*g/ml) and then stored in microtubules at -80°C. Tumor necrosis factor- (TNF-) *α*, interleukin- (IL-) 10, IL-6, and IL-1 were measured. An enzyme-linked immunosorbent assay (ELISA) was performed in accordance with the manufacturer's instructions. Measurements were obtained with an automated microplate reader (Multiskan MK3; Thermo Fisher Scientific, Inc., Waltham, MA) at an optical absorbance value of 450 nm.)

### 2.4. Immunofluorescence

The cell suspension for immunofluorescence was injected into the corresponding experimental wells and cultured in 5% CO_2_ at 37°C for 4 h to make the cells adhere to the plate. The nonadhered cells were washed with PBS (5 min × 3) and then fixed in newly prepared 4% paraformaldehyde for 15 min at room temperature. After fixation, the cells were washed with PBS and then permeabilized with HEPES Triton buffer (20 mm HEPES, 300 mM sucrose, 50 mM NaCl, 3 mM MgCl_2_, 0.5% Triton X-100, pH 7.4) for 1 h. After rinsing with PBS, the cells were blocked with 10% BSA (PBS dilution) at room temperature for 1 h and then incubated with CD14 (rabbit monoclonal antibody; Santa Cruz Biotechnology) and TLR4 (mouse monoclonal antibody; Santa Cruz Biotechnology) overnight at 4°C (all antibodies in 10% BSA/PBS were 1 : 50). The next day, the cells were washed with PBS (5 min × 3) and then cultured in the dark for 2 h at room temperature with fluorescein-conjugated goat anti-rabbit IgG and rhodamine combined with goat anti-mouse IgG (1 : 200 in PBS; Zhongshan Jinqiao Biotechnology Co., Ltd., Beijing, China). The negative control was created by replacing the first or second antibody with species- and isotype-matched irrelevant antibodies. For blank controls, the first or second antibody was replaced with PBS. Slides were examined with a fluorescence microscope (Leica-DM-4000B; Leica, Wetzlar, Germany). A single masked observer randomly selected five high-power fields to analyze each stain. Images were captured using an inverted confocal laser scanning microscope (Leica-DM-IRE2; Leica).

### 2.5. Statistical Analysis

Statistical analysis was performed using SPSS 22.0 (SPSS Inc., Chicago, IL) software. For multiple comparisons, different groups were analyzed using one-way analysis of variance (ANOVA), followed by Bonferroni test. A *P* value of less than or equal to 0.05 was considered significant. Fluorescence analysis and cell counts were completed using ImageJ (National Institutes of Health) software.

## 3. Results

### 3.1. Cell Identification

**Figure 1:**
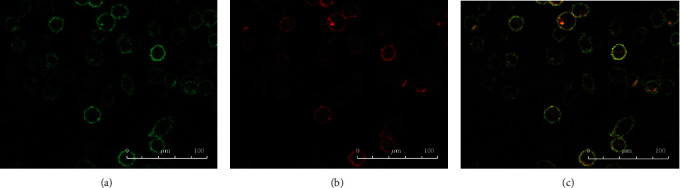
Immunofluorescence staining of TLR4 and CD14. (a) CD14 identifies monocytes. The cells were nearly round. (b) Some monocytes expressed TLR4 without LPS stimulation; TLR4 mainly expressed on the cell membrane. (c) Without LPS stimulation, some monocytes coexpress TLR4 and CD14.

The isolated monocytes were identified by CD14 immunofluorescence staining, and the cells appeared approximately round (Figure 1).

### 3.2. Changes in Cell Morphology after LPS Stimulation

**Figure 2:**
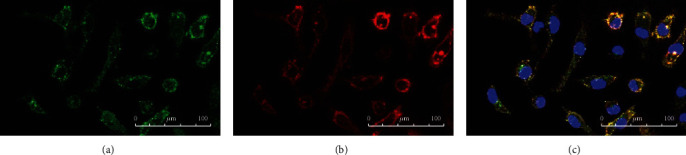
Immunofluorescence of TLR4 and CD14 in the ANA+ group after LPS stimulation. (a) Monocytes were identified by CD14. Some cells changed from round to fusiform. (b) TLR4 was mainly expressed in the cell membrane. (c) With LPS stimulation, the mononuclear cells coexpressed TLR4 and CD14.

**Figure 3:**
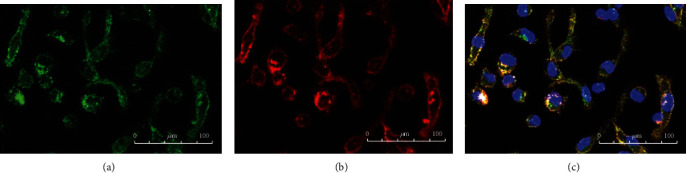
Immunofluorescence of TLR4 and CD14 in the ANA- group after LPS stimulation. (a) Monocytes were identified by CD14. Some cells changed from round to fusiform. (b) TLR4 was mainly expressed in the cell membrane. (c) Under LPS stimulation, mononuclear cells coexpressed TLR4 and CD14.

**Figure 4:**
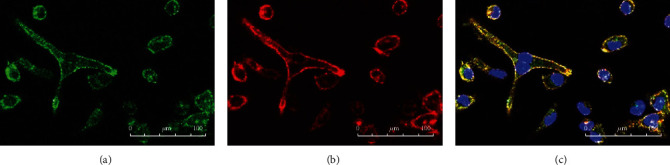
Immunofluorescence of TLR4 and CD14 in the control group after LPS stimulation. (a) Monocytes were identified by CD14. A few cells changed. (b) TLR4 was mainly expressed on the cell membrane. (c) TLR4 and CD14 were expressed in mononuclear cells without LPS stimulation.

**Figure 5:**
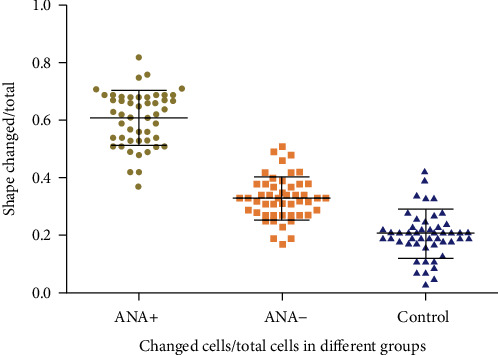
Changed cells/total cells in different groups. This rate in the ANA+ group was significantly higher than that in the other two groups (*F* = 295.812, *P* = 2.9423 × 10*e* − 15) Data was expressed as mean ± SD (*n* = 10 for each group, five visual fields were selected for each object to get the number of deformed cells, repeated three times).

After LPS stimulation, we found that part of cells changed in each group, and this mainly manifested as cell deformation and area enlargement (see Figures 2–4). Since the number of individuals in the view field was different, we believed that it was more meaningful to count the ratio of changed/total than simply calculating the changed cells in the view field. Therefore, we used changed/total cells to compare the differences among groups. We found that the deformation rate of the ANA+ group was significantly higher than that of the other two groups (*F* = 295.812, *P* = 2.9423 × 10*e* − 15) (see Figure 5). Due to the limitation of blood collection, we only analyzed the changes in cell morphology (peak cytokine) at 6 h after LPS stimulation.

### 3.3. ELISA

**Figure 6:**
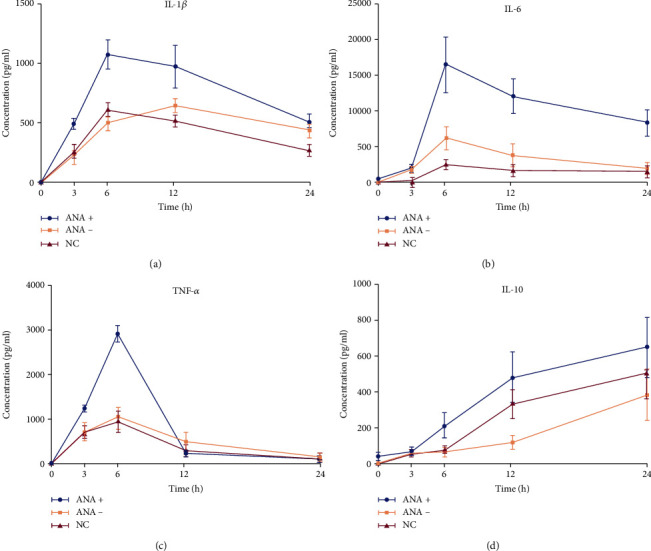
Changes in the cytokines in each group after LPS stimulation. Six hours after stimulation, IL-1*β*, IL-6, and TNF-*α* reached their peaks and then decreased. Within 24 h, IL-10 showed an upward trend.

In this study, we found that IL-10 kept rising within 24 hours of LPS stimulation. In contrast, TNF-*α*, IL-1*β*, and IL-6 reached their peaks at 6 h after LPS stimulation (Figure 6). The initial concentration of IL-1*β* for all groups was not statistically significant (*P* = 0.293). The differences between groups at other time points (3, 6, 12, and 24) were statistically significant (*P* = 2.1505*e* − 15, *P* = 5.1213*e* − 23, *P* = 3.216*e* − 17, and *P* = 1.1331*e* − 14, respectively). For IL-6, 0 hours after LPS stimulation, there was no statistically significant difference between groups (*P* = 0.150), and at the other time points (3, 6, 12, and 24), the differences between groups were statistically significant (*P* = 5.8878*e* − 17, *P* = 1.9211*e* − 27, *P* = 2.2502*e* − 27, and *P* = 3.3387*e* − 28, respectively). Comparison of TNF-*α* levels in each group showed that there was no statistically significant differences at 0 hours after LPS stimulation (*P* = 0.114) in each group, and at the other time points (3, 6, 12, and 24) between the groups, the differences were statistically significant (*P* = 1.6492*e* − 13, *P* = 2.1564*e* − 21, *P* = 4.3904*e* − 9, and *P* = 0.000084, respectively). The analysis of IL-10 showed that the differences between the groups at each time (0, 3, 6, 12, and 24) were statistically significant (*P* = 1.0325*e* − 7, *P* = 0.024, *P* = 5.3786*e* − 15, *P* = 8.0653*e* − 14, and *P* = 9.0477*e* − 9, respectively).

## 4. Discussion

JIA shows the most common identifiable systemic association with uveitis. Oligoarticular onset of arthritis, younger age at onset, and the presence of circulating ANAs are the main risk factors for the development of uveitis [9, 10]. In recent years, the prognosis of JIAU has been much better than that in the past because of the maturity of the therapeutic schedule. However, due to the young age of onset, and because the symptoms are not obvious, consequently, it is easy for parents to ignore this disease, especially in areas where children's health care is insufficient. In this study, some children went to the ophthalmology clinic due to severe complications, and the prognosis of this population was poor. Therefore, it is necessary to explore the possible pathogenesis of JIAU and pay close attention to high-risk groups in order to seek better treatments.

In the outpatient, we found that most of the young JIAU patients were ANA+, and these patients often came to the ophthalmology department for serious ocular complications. Surprisingly, with the control of systemic and ocular inflammation, the titer of antinuclear antibodies decreased and even became negative. We do not know why this happens, and there was no relevant report. Coincidentally, Rapp and his colleagues reported that after long-term observation of some patients with systemic lupus erythematosus (SLE), ANA became negative after treatment [11]. Therefore, we wondered whether it was more serious for the ANA titer to be positive in these types of disease. Of course, ANA positivity is related to many diseases, such as connective tissue disease, Sjogren's syndrome, scleroderma, autoimmune diseases, hepatitis, primary biliary cirrhosis, ulcerative colitis, and autoimmune thyroiditis [12–18]. We do not believe that ANA is a specific marker of JIAU, and whether systemic arthritis and ocular inflammation are improved is not determined by the ANA titer. However, our inference is that ANA plays a special role in the pathogenesis of JIAU.

JIAU is a chronic inflammatory disease, mainly manifested as anterior uveitis. The main cause of poor vision is frequent acute attacks that cause serious ocular complications. Our previous studies on the acute animal models found that LPS-Toll-like receptor 4 (TLR4) signal transduction on macrophages plays an important role in the acute episode of uveitis [6, 7, 19]. Were there any differences in the LPS-TLR4 signaling pathway between ANA+ and ANA- patients? In this study, we isolated and cultured PBMCs from JIAU patients and observed the difference in their response to LPS. We measured cytokines and observed changes in cell morphology to explore the differences in the response to LPS between ANA+ and ANA- groups of patients with JIAU.

In this work, we found that, unlike IL-10, cytokines IL-1*β*, IL-6, and TNF-*α* in all groups reached their peak 6 h after LPS stimulation and then decreased. IL-10 is an effective anti-inflammatory factor, which can inhibit Th1 cells from producing inflammatory factors. Labeled as a cytokine synthesis inhibitor [20], IL-10 also prevents disease by inhibiting activation of macrophages and dendritic cells (DCs), thereby suppressing the immune response to pathogens and microorganisms [21]. In this research, IL-10 continued to increase within 24 hours of LPS stimulation, while other cytokines decreased to a lower level after 24 h. Studies on animal models of acute uveitis had found that inflammation been significantly reduced 24 hours after LPS stimulation. IL-1*β*, IL-6, and TNF-*α* in each group, which increased rapidly, reached their peak 6 h after LPS stimulation and then decreased. The cytokines in the ANA+ group rose fiercely, indicating that these patients responded quickly and strongly to LPS. Although the growth trend was similar, the response of the ANA- group was slower, and the response of the control group was relatively flat. Using immunofluorescence staining technology, we first used CD14, a monocyte marker, to identify the cultured cells and observed the changes in the cell shape after LPS stimulation (in order to show the changes in cell shape more intuitively, we labeled TLR4, a protein mainly expressed on the cell membrane [22]). Our conclusion was that the ratio of morphologically changed cells/total cells was the highest in the ANA+ group. The changed cells are mainly manifested as an area increase and extended pseudopods. We believed this was a manifestation of monocyte activation, which happened to be consistent with the report of Gordon [23].

Nucleic acids, nucleosomes, phospholipids, and several nuclear and nucleolar proteins are the key measurement indicators for ANA. These autoantigens are normally hidden, but when cells die, especially when they undergo apoptosis, autoantigens are exposed to antigen-presenting cells [24, 25]. Why does positivity for ANAs predict a higher risk of JIAU? As we know, JIA occurs due to an immune self-tolerance breakdown. Some studies have suggested that the occurrence of JIA is related to major histocompatibility complex class II alleles [26–28]; this suggests a critical role for CD4+ T helper (Th) cells. Synovial fluid from inflamed joints in children with arthritis shows an abnormal ratio of Th17 to regulatory T cell subsets, and the number of Th17 cells was associated with arthritis severity [29]. Systemic JIA is a special subtype driven mainly by innate immunodeficiency [30]. Linking our previous studies [5–7, 19] to animal models, we concluded that ANA positivity increases over activation of the antigen-presenting cellular immune state in iris tissues, initiating the immune response and causing inflammation.

During the collection of cases, we found that children in the ANA+ group had earlier onset compared with the ANA- group. However, there was no significant difference in the onset age between the two groups (*P* = 0.091). The possible reason for the earlier onset was that ANA positivity increases the sensitivity of monocytes to LPS, activates the transduction of LPS-TLR4 on monocytes, produces cytokines, and triggers inflammation. Of course, this is only a preliminary speculation, and its specific mechanisms and cell functions need further research. The main limitation of our study was that the parents of the children did not agree to collect a large amount of venous blood for experimental research, which caused a small amount of extracted cells. In order to minimize the impact on the experimental results, all patients enrolled in this study were in remission and used the same drugs. Of course, the number of monocytes extracted from the subject would vary with gender, age, and even the operators. For the purpose of minimizing errors, we strictly controlled the number of cells that each subject entered the experiment. In this work, due to the insufficient number of cells, we only selected 6 h (peak of cytokine) after LPS stimulation and observed the cell morphology by immunofluorescence staining. In the future, if we can collect more blood, we will evaluate the changes in cell morphology at different times. We hope that this article can be used as a preliminary exploration of cytokine differences between patients with different ANAs and JIAU.

In this study, with LPS stimulation, the ratio of changed cells/total cells in the ANA+ group was significantly higher than that in the other groups, indicating that ANA positivity may increase the sensitivity of monocytes to LPS stimulation. The specific mechanism has not been reported thus far, and this is also the goal of our future research.

## Figures and Tables

**Table 1:** Information of the ANA+ patients.

No.	Age	Gender	JIA onset	JIAU onset	Ocular complication
1	2	F	1	1.5	Cataract
					Posterior synechiae

2	2	F	1.5	1.5	Cataract
					Posterior synechiae

3	5	F	2	3	Cataract

4	7	M	5	5	Band keratopathy
					Cataract

5	9	F	6	7	Cataract
					Posterior synechiae
					Glaucoma

6	9	M	7	7	Cataract

7	10	F	7	8	Band keratopathy
					Cataract

8	11	F	8	9	Cataract
					Posterior synechiae
					Glaucoma

9	14	M	9	11	Band keratopathy
					Cataract

10	16	M	11	11	Cataract

**Table 2:** Information of the ANA- patients.

No.	Age	Gender	JIA onset	JIAU onset	Ocular complication
1	7	M	6	6	Cataract
					Posterior synechiae

2	7	F	5	5	Cataract

3	8	F	6	7	Cataract
					Posterior synechiae

4	9	M	7	7	Posterior synechiae
					Cataract

5	12	F	9	11	Cataract
					Posterior synechiae

6	12	F	8	8	Cataract
					Posterior synechiae
					Glaucoma

7	13	M	9	10	Posterior synechiae
					Cataract
					Glaucoma

8	14	F	10	12	Cataract

9	14	M	9	12	Cataract

10	16	F	12	12	Cataract

## Data Availability

The data used to support the findings of this study are included within the article.

## References

[1] Barut K., Adrovic A., Şahin S., Kasapçopu Ö. (2017). Juvenile idiopathic arthritis. *Balkan Medical Journal*.

[2] Heiligenhaus A., Minden K., Föll D., Pleyer U. (2015). Uveitis in juvenile idiopathic arthritis. *Deutsches Ärzteblatt International*.

[3] Kotaniemi K., Kautiainen H., Karma A., Aho K. (2001). Occurrence of uveitis in recently diagnosed juvenile chronic arthritis: a prospective study. *Ophthalmology*.

[4] Manners P. J., Bower C. (2002). Worldwide prevalence of juvenile arthritis why does it vary so much?. *The Journal of Rheumatology*.

[5] Chen W., Hu X., Zhao L., Li S., Lu H. (2009). Expression of toll-like receptor 4 in uvea-resident tissue macrophages during endotoxin-induced uveitis. *Molecular Vision*.

[6] Yang S., Lu H., Wang J., Qi X., Liu X., Zhang X. (2012). The effect of toll-like receptor 4 on macrophage cytokines during endotoxin induced uveitis. *International Journal of Molecular Sciences*.

[7] Li S., Lu H., Hu X., Chen W., Xu Y., Wang J. (2010). Expression of TLR4-MyD88 and NF-*κ*B in the iris during endotoxin-induced uveitis. *Mediators of Inflammation*.

[8] Antonakos N., Tsaganos T., Oberle V. (2017). Decreased cytokine production by mononuclear cells after severe gram-negative infections: early clinical signs and association with final outcome. *Critical Care*.

[9] Clarke S. L. N., Sen E. S., Ramanan A. V. (2016). Juvenile idiopathic arthritis-associated uveitis. *Pediatric Rheumatology*.

[10] Tappeiner C., Klotsche J., Sengler C. (2018). Risk factors and biomarkers for the occurrence of uveitis in juvenile idiopathic arthritis. Data from the inception cohort of newly diagnosed patients with juvenile idiopathic arthritis study. *Arthritis & Rheumatology*.

[11] Rapp C. A., Berner B., Müller G. A., Reuss-Borst M. A. (2002). Krankheitsaktivität und chronische organschäden bei patienten mit systemischem lupus erythematosus (SLE) im langzeitverlauf. *Zeitschrift für Rheumatologie*.

[12] Barahona-Garrido J., Camacho-Escobedo J., García-Martínez C. I., Tocay H., Cabiedes J., Yamamoto-Furusho J. K. (2009). Antinuclear antibodies: a marker associated with steroid dependence in patients with ulcerative colitis. *Inflammatory Bowel Diseases*.

[13] Campanilho-Marques R., Bogas M., Ramos F., Santos M. J., Fonseca J. E. (2014). Prognostic value of antinuclear antibodies in juvenile idiopathic arthritis and anterior uveitis. Results from a systematic literature review. *Acta Reumatologica Portuguesa*.

[14] Radic M., Herrmann M., van der Vlag J., Rekvig O. P. (2011). Regulatory and pathogenetic mechanisms of autoantibodies in SLE. *Autoimmunity*.

[15] Segni M., Pucarelli I., Truglia S., Turriziani I., Serafinelli C., Conti F. (2014). High prevalence of antinuclear antibodies in children with thyroid autoimmunity. *Journal of Immunology Research*.

[16] Thomson A. M., West D. C. (1990). Factors affecting slow regular firing in the suprachiasmatic nucleus in vitro. *Journal of Biological Rhythms*.

[17] Weiss J. E. (2012). Pediatric systemic lupus erythematosus: more than a positive antinuclear antibody. *Pediatrics in Review*.

[18] Yu C., Gershwin M. E., Chang C. (2014). Diagnostic criteria for systemic lupus erythematosus: a critical review. *Journal of Autoimmunity*.

[19] Wang J., Lu H., Hu X. (2011). Nuclear factor translocation and acute anterior uveitis. *Molecular Vision*.

[20] Sziksz E., Pap D., Lippai R. (2015). Fibrosis related inflammatory mediators: role of the IL-10 cytokine family. *Mediators of Inflammation*.

[21] Kasama T., Strieter R. M., Lukacs N. W., Lincoln P. M., Burdick M. D., Kunkel S. L. (1995). Interleukin-10 expression and chemokine regulation during the evolution of murine type II collagen-induced arthritis. *The Journal of Clinical Investigation*.

[22] Patra M. C., Choi S. (2016). Recent progress in the development of toll-like receptor (TLR) antagonists. *Expert Opinion on Therapeutic Patents*.

[23] Gordon S. (2002). Pattern recognition receptors: doubling up for the innate immune response. *Cell*.

[24] Casciola-Rosen L. A., Anhalt G., Rosen A. (1994). Autoantigens targeted in systemic lupus erythematosus are clustered in two populations of surface structures on apoptotic keratinocytes. *The Journal of Experimental Medicine*.

[25] Rahman A., Isenberg D. A. (2008). Systemic lupus erythematosus. *The New England Journal of Medicine*.

[26] Vehe R. K., Begovich A. B., Nepom B. S. (1990). HLA susceptibility genes in rheumatoid factor positive juvenile rheumatoid arthritis. *The Journal of Rheumatology*.

[27] de Silvestri A., Capittini C., Poddighe D. (2017). HLA-DRB1 alleles and juvenile idiopathic arthritis: diagnostic clues emerging from a meta-analysis. *Autoimmunity Reviews*.

[28] Hersh A. O., Prahalad S. (2015). Immunogenetics of juvenile idiopathic arthritis: a comprehensive review. *Journal of Autoimmunity*.

[29] Nistala K., Moncrieffe H., Newton K. R., Varsani H., Hunter P., Wedderburn L. R. (2008). Interleukin-17-producing T cells are enriched in the joints of children with arthritis, but have a reciprocal relationship to regulatory T cell numbers. *Arthritis and Rheumatism*.

[30] Pardeo M., Bracaglia C., De Benedetti F. (2017). Systemic juvenile idiopathic arthritis: new insights into pathogenesis and cytokine directed therapies. *Best Practice & Research Clinical Rheumatology*.

